# Consensus-based statements for the management of mitochondrial stroke-like episodes

**DOI:** 10.12688/wellcomeopenres.15599.1

**Published:** 2019-12-13

**Authors:** Yi Shiau Ng, Laurence A. Bindoff, Gráinne S. Gorman, Rita Horvath, Thomas Klopstock, Michelangelo Mancuso, Mika H. Martikainen, Robert Mcfarland, Victoria Nesbitt, Robert D. S. Pitceathly, Andrew M. Schaefer, Doug M. Turnbull

**Affiliations:** 1 Wellcome Centre for Mitochondrial Research, Newcastle University, UK, Newcastle upon Tyne, Tyne and Wear, NE2 4HH, UK; 2 Directorate of Neurosciences, Newcastle Upon Tyne Hospitals NHS Trust, Newcastle upon Tyne, Tyne and Wear, NE1 4LP, UK; 3 NHS Highly Specialised Service for Rare Mitohcondrial Disorders, Royal Victoria Infirmary, Newcastle upon Tyne, UK; 4 Department of Clinical Medicine, University of Bergen, Bergen, Norway; 5 Department of Neurology, Haukeland University Hospital, Bergen, Norway; 6 Department of Clinical Neurosciences, University of Cambridge, Cambridge, UK; 7 Department of Neurology, Friedrich-Baur-Institute, University Hospital of the Ludwig-Maximilians-Universität München, Munich, Germany; 8 German Center for Neurodegenerative Diseases (DZNE), Munich, Germany; 9 Munich Cluster for Systems Neurology (SyNergy), Munich, Germany; 10 Department of Clinical and Experimental Medicine, Neurological Clinic, University of Pisa, Pisa, Italy; 11 Division of Clinical Neurosciences, University of Turku and Turku University Hospital, Turku, Finland; 12 Great North Children Hospital, Newcastle upon Tyne Hospitals NHS Foundation Trust, Newcastle upon Tyne, UK; 13 Department of Paediatrics, The Children's Hospital, Oxford, UK; 14 NHS Highly Specialised Service for Rare Mitochondrial Disorders,, Nuffield Dept Women’s & Reproductive Health, The Churchill Hospital, Oxford, UK; 15 MRC Centre for Neuromuscular Diseases, UCL Queen Square Institute of Neurology and National Hospital for Neurology and Neurosurgery, London, UK; 16 NHS Highly Specialised Service for Rare Mitochondrial Disorders, Centre for Neuromuscular Diseases, The National Hospital of Neurology and Neurosurgery, London, UK

**Keywords:** MELAS, m.3243A>G, POLG, epilepsy, status epilepticus, encephalopathies, stroke, neurodegenerative disorders (other than dementia), antiepileptic drugs

## Abstract

**Background: **Focal-onset seizures and encephalopathy are prominent features of a stroke-like episode, which is a severe neurological manifestation associated with subtypes of mitochondrial disease. Despite more than 30 years of research, the acute treatment of stroke-like episodes remains controversial.

**Methods: **We used the modified Delphi process to harness the clinical expertise of a group of mitochondrial disease specialists from five European countries to produce consensus guidance for the acute management of stroke-like episodes and commonly associated complications.

**Results: **Consensus on a new definition of mitochondrial stroke-like episodes was achieved and enabled the group to develop diagnostic criteria based on clinical features, neuroimaging and/or electroencephalogram findings. Guidelines for the management of strokelike episodes were agreed with aggressive seizure management strongly recommended at the outset of stroke-like episodes.

**Conclusions: **Our consensus statement defines stroke-like episodes in terms of an epileptic encephalopathy and we have used this to revise both diagnostic criteria and guidelines for management. A prospective, multi-centre, randomised controlled trial is required for evaluating the efficacy of any compound on modifying the trajectory of stroke-like episodes.

## Introduction

Stroke-like episodes are a well-recognised feature of some forms of mitochondrial disease and were first reported in association with the syndrome mitochondrial encephalomyopathy, lactic acidosis and stroke-like episodes (MELAS) ^1^. These episodes are characterised by headache, nausea and vomiting, encephalopathy, focal-onset seizures (with or without associated focal neurological deficits) and cortical and sub-cortical signal abnormalities not confined to vascular territories. While originally described in individuals under the age of 40 ^2^, late-onset presentation is increasingly recognised ^3– 5^. The first genetic defect linked to MELAS was a heteroplasmic mitochondrial DNA (mtDNA) mutation, m.3243A>G ^6^, and this accounts of ~80% of the cases reported in the early literature ^7^. Subsequently, other rarer mtDNA mutations such as m.3271T>C ( *MT-TL1*) and m.13513G>A ( *MT-ND5*) ^8^ have been linked to MELAS and more recently, recessive *POLG* mutations have emerged as an important cause of refractory seizures and stroke-like episodes ^9– 11^. The underlying pathophysiological cerebral dysfunction appears to be the same in both mtDNA (e.g. m.3243A>G) and nuclear gene ( *POLG*) induced stroke-like episodes ^12^.

The underlying mechanism of stroke-like episodes remains unclear, but several hypotheses have attempted to elucidate the pathophysiology based on the laboratory, clinical, radiological and neuropathological observations. The vascular theory emphasises the potential role of mitochondrial proliferation in the smooth muscle layer of small arteries and arterioles, causing impaired autoregulation that results in ischaemia and development of stroke-like lesions ^13^. Further, several studies reported a link between nitric oxide deficiency, low plasma levels of arginine ^14^ and citrulline ^15^ and the pathogenesis of stroke-like episodes ^16^. However, stroke-like lesions typically are not confined to a single vascular territory, and the hypoperfusion to the stroke-like lesions has not been convincingly demonstrated during the acute stroke-like episodes ^17– 19^. Moreover, no evidence of ischaemia was identified, in a post-mortem study in patients with *POLG*-disease ^20^. In contrast, the theory of neuronal hyper-excitability, suggests that stroke-like episodes are mediated by ictal activity. This was based on the observation that seizures are commonly present at the outset of stroke-like episodes ^21, 22^, and supported by recent neuropathological findings showing severe mitochondrial complex I defects and preferential loss of inhibitory inter-neurons that potentially could lead to neuronal hyper-excitability ^23^.

Limitations in our understanding of the mechanisms involved in this disease is also reflected in the controversies surrounding the management of stroke-like episodes. Some clinicians advocate the use of intravenous L-arginine ^24, 25^ based on the theory that nitric oxide is involved, and the findings from single, small open-label trials ^14^ and other anecdotal case reports ^26^. Others suggest that seizures are the proximate cause and that seizure management and other supportive measures are crucial ^27– 29^. The long-term consequence of recurrent stroke-like episodes is cognitive impairment due to neurodegeneration which marked brain atrophy has been frequently observed in the neuroimaging of patients affected by stroke-like episodes and seizures ^30^, and necrotic lesions in cortices and significant neuronal loss have been identified in neuropathological studies ^10, 31^.

While all agree that patients presenting with the stroke-like episodes require prompt and effective treatment, controversies surrounding mechanisms has led to confusion concerning how best to approach management. We sought to address the areas of uncertainties in clinical practice where evidence is either inconclusive or absent, by harnessing the group opinion of experts from multiple medical centres in the UK and Europe using the modified Delphi method. The consensus-based best practice recommendations that resulted from this workshop aim to increase the awareness of stroke-like episodes among health care personnel and improve outcomes for patients by providing standardised care for patients with mitochondrial stroke-like episodes. The target audience for this document includes neurologists, paediatricians, emergency medicine specialists, general and acute physicians, primary care clinicians, radiologists and other clinicians who investigate and manage mitochondrial diseases.

## Methods

The Delphi technique was a research methodology originally designed to study the forecast of technological events by eliciting and refining group opinion of experts several decades ago ^32^. Modified Delphi method has subsequently been developed and extensively adopted in different disciplines of medical field, in situations where there is little evidence, where the data are incomplete or not readily applicable to the area of interest, or where the data are extensive but conflicting ^33^. The key distinction between the original and modified Delphi methods is the incorporation of face-to-face meeting in the process of reaching consensus in the latter ^34^.

We applied the modified Delphi method consisted of two rounds of email questionnaire (the first and second questionnaires were distributed on 16 ^th^ February 2018 and 28 ^th^ March 2018, respectively) and a face-to-face meeting in this work. A panel comprising leading clinical specialists in mitochondrial disease from the UK (n=8), Norway (n=1), Italy (n=1), Germany (n=1) and Finland (n=1) was formed. A questionnaire with a list of statements was devised based on the Newcastle best practice guidelines ( http://www.newcastle-mitochondria.com/guidelines/) and review of current literature by a facilitator (Y.S.N.), and distributed to the panel participants before the meeting. The questionnaire contained 15 sections and 88 questions ( *Extended data*, Supplemental Table 1 ^35^). For each statement, participants voted using a five-point Likert scale to indicate the level of agreement (1 = strongly disagree, 2 = disagree, 3 = neither disagree nor agree, 4 = agree, 5 = strongly agree). The responses were collected, analysed anonymously and fed back to the panel members. An agreement was reached when both more than 70% of scores were ≥ 4 and the mean score was ≥ 4, as previously defined in our other work ^36^.

The panel members convened at a workshop held in Newcastle between 1 ^st^ and 2 ^nd^ of March 2018. Case studies of mitochondrial stroke-like episodes were presented and panel members exchanged their thoughts and clarify reasons for disagreements in the pre-meeting questionnaire. The second questionnaire, including new questions, was devised and voted by the panel members at the workshop. The second questionnaire contained 15 sections and 77 questions; the numbers of questions that were removed or rephrased were summarised in the *Extended data*, Supplemental Table 2 ^35^. Consensus-based recommendations were based on responses in which more than 70% of scores were ≥ 4 and the mean score was ≥ 4.

Two representatives from the patient and charity organisations (Lily Foundation and International Mito Patients (IMP)) participated in the discussion at the workshop but were not expected to complete the questionnaires.

All participants voluntarily accepted the invitation to take part in this study and agreed for the dissemination of the findings. Ethical approval was not required as the study only involved the general discussion on clinical practice. Individual responses to two questionnaires were anonymised.

## Results and Discussion

**Figure 1.**
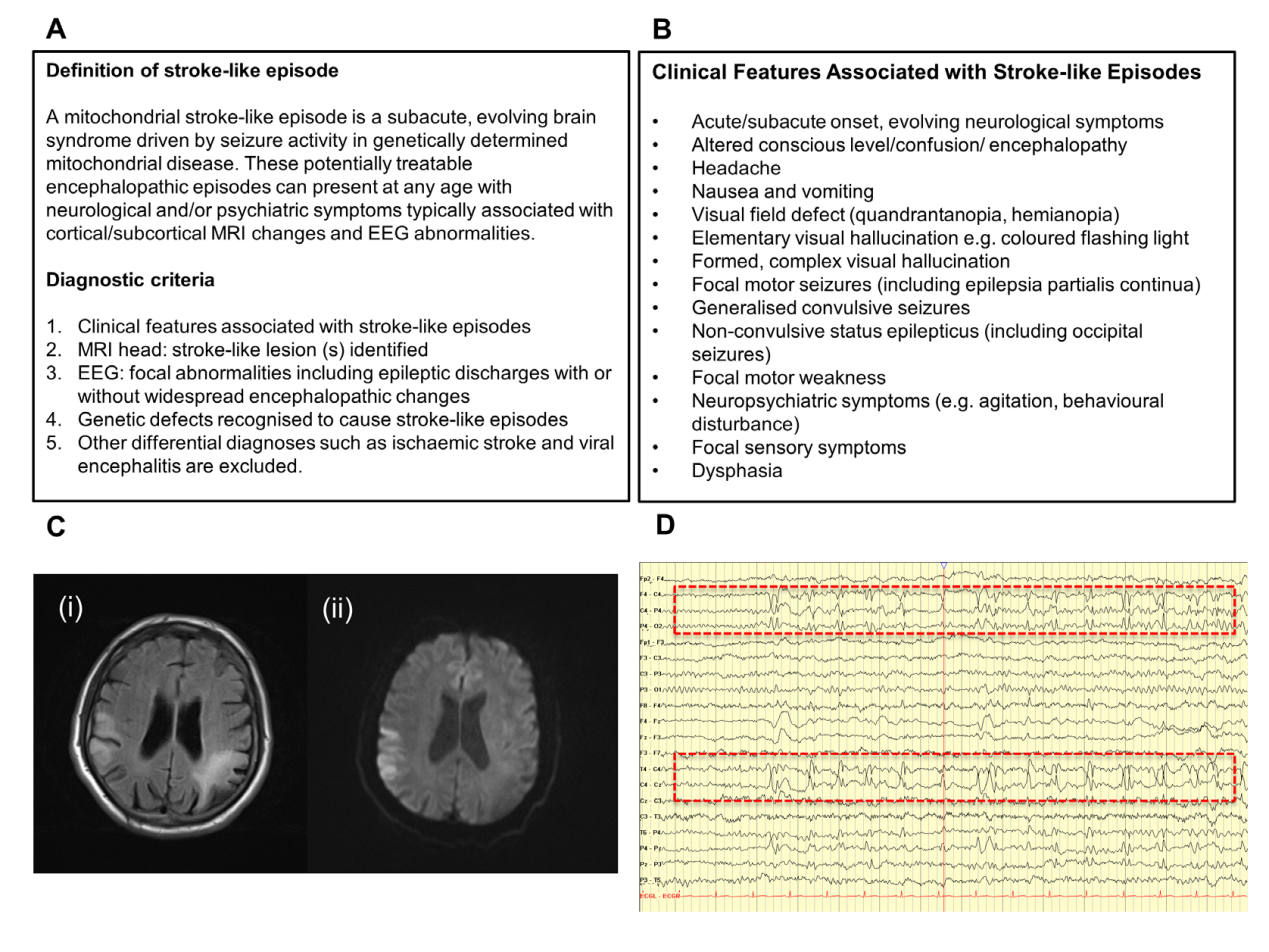
Definition, clinical features and diagnostic criteria of stroke-like episodes. ( **A**) Consensus-based definition of mitochondrial stroke-like episode and diagnostic criteria. ( **B**) A list of neurological signs and symptoms associated with stroke-like episodes. ( **C**) MRI head FLAIR-sequence (i) shows typical stroke-like lesions (confluent cortical and subcortical hyperintensities) involving bilateral parietal lobes with restricted diffusion demonstrated in the right parietal lobe (ii). This patient who has the m.3243A>G-related MELAS syndrome presented with subacute-onset intermittent speech arrest, confusion and focal motor seizures affecting left arm and leg. ( **D**) The electroencephalogram (EEG) of the same patient shows a well-formed and symmetrical 8–9Hz alpha rhythm intermixed with a large amount of diffuse theta wave in the temporal areas. A persistent sharp/spike and slow wave focus is seen over the right central electrodes (dotted-line red boxes), corresponding with the stroke-like lesion in the right parietal area identified on the MRI scan.

These recommendations cover both the acute management of stroke-like episodes and management of the immediate sequelae ( *Extended data*, Supplemental Table 3 ^35^). Individual responses to pre- and post-workshop questionnaires are available as *Underlying data*. The consensus opinion of the expert panel was that stroke-like episodes reflected seizure activity and not ischaemia (the agreed definition is given in Figure 1A). While our recommendations reflect this, we would not deflect physicians from treating other potential aetiologies as long as seizures treatment forms a major part of the management focus.

### Recognition of stroke-like episodes

Patients with stroke-like episodes and their caregivers frequently encounter challenges and difficulties when accessing local health services due to the lack of expertise in mitochondrial disorders. To improve the care and outcome, patients, their caregivers and attending clinicians should be informed about the nature and consequences of these episodes to ensure that all can recognise the clinical features from the outset ( Figure 1B). It is agreed that the diagnosis of a stroke-like episode requires the combination of clinical assessment, MRI head and electroencephalogram (EEG) ( Figure 1C, D). We advocate that an emergency care plan should be provided to all patients who have already suffered from stroke-like episodes, and their caregivers. For carriers of pathogenic variants known to cause stroke-like episodes, they should be provided with an alert card that facilitates early recognition of clinical symptoms associated with stroke-like episodes.

Given the devastating nature and complexity of a mitochondrial stroke-like episode, we recommend that all cases are discussed with, or referred to, a mitochondrial disease specialist in the acute setting.

### Assessment and investigations

It is crucial to obtain a detailed history from the patient or caregiver when dealing with a patient presenting with a suspected stroke-like episode. A hyper-acute onset of focal neurological deficit, particularly pure motor weakness (facial weakness or hemiparesis) that evolves rapidly within minutes is unusual for a mitochondrial stroke-like episode and more suggestive of a vascular stroke. Clinicians should refer to their national guidelines for the investigation and treatment of vascular stroke.

For any individual who presents with complex visual symptoms, perceptive problems and hearing disturbances persisting for hours or days before admission, the attending clinician should consider the possibility of a mitochondrial stroke-like episode. The clinical assessment should comprise a systemic enquiry and general physical examination to identify potential triggers such as infection, gut dysmotility, dehydration, prolonged fasting and non-adherence to the anti-epileptic drug(s). A full neurological examination with particular attention to the level of consciousness, visual field testing, and evaluation of speech and signs of apraxia should be performed.

For any suspected stroke-like episodes in patients who are known to harbour primary mtDNA or recessive *POLG* pathogenic variants, the following investigations should be considered ( Table 1):

- MRI head
  - Proposed protocol: T1, T2, FLAIR, DWI and ADC
  - If MRI head is contra-indicated, CT head can be performed
- Electroencephalogram (EEG)
- Blood and laboratory tests
- Chest radiograph (if aspiration pneumonia suspected)
- Abdominal radiography (if intestinal pseudo-obstruction is suspected)
- Electrocardiogram (ECG)

**Table 1.** Recommended investigations for patients presenting with (suspected) stroke-like episode. MRI head and EEG are essential for confirming the diagnosis. CT head can be performed if there is any contraindication for performing MRI head.

Blood and laboratory tests
❖ Full blood count
❖ Urea, creatinine and electrolytes
❖ Liver function test (LFT)
❖ Random glucose
❖ Serum lactate (without tourniquet applied)
❖ C-reactive protein (CRP)
❖ Urinalysis and urine culture (septic screen)
❖ Anti-epileptic drug level (e.g. phenytoin, carbamazepine, phenobarbitone) if applicable
❖ Coagulation screen (for patients with *POLG* pathogenic variants)
❖ Creatine kinase (CK)
❖ HbA1c (for known diabetic)
❖ Blood culture (septic screen)
❖ Arterial blood gas (for pH if hyperlactaemia is present or respiratory insufficiency is suspected)
**MRI head** (minimal sequences should include T1, T2, FLAIR, DWI and ADC) **Electroencephalogram** (EEG) **Chest radiography** (if aspiration pneumonia is suspected) **Abdominal radiography** (if intestinal pseudo-obstruction is suspected) **12-lead electrocardiogram** (ECG)

### Management of seizures in stroke-like episodes

Focal seizures (including non-motor seizures such as occipital seizures) with or without evolution to bilateral convulsive seizures are frequently associated with the stroke-like episodes. Patients with previous history of stroke-like episodes who report symptoms suggestive of a new episode or seizure, and are not in hospital, should be considered for early commencement of benzodiazepine. Once in hospital patients should be treated urgently with an intravenous (IV) anti-epileptic drug (AED):

- Intravenous levetiracetam (20–40 mg/kg, max 4500 mg) is recommended, but phenytoin (15–20 mg/kg with cardiac monitoring), phenobarbitone (10–15 mg/kg with respiratory monitoring) or lacosamide (200–400 mg) can be used.

Sodium valproate is contra-indicated for patients with recessive *POLG* pathogenic variants, and should also be avoided in patients with mitochondrial epilepsy caused by other genotypes if an alternative drug is available.

Non-availability of EEG or MRI head should not deter or delay treatment of patients with (suspected) stroke-like episodes. Nasogastric tube (NGT) insertion should be considered early for administering usual AEDs and other medications if oral route is not reliable due to encephalopathy or vomiting. In cases of stroke-like episode needing IV AEDs, the patient should be maintained on an AED regime.

Should the patient develop generalised, convulsive status epilepticus, further management such as escalation to intensive care setting should follow the local status epilepticus guidelines (e.g. NICE Clinical Guidelines 137, SIGN guidelines 143, EFNS guidelines 2010), except sodium valproate should be avoided.

### Management of stroke-like episodes at the intensive care unit

We recommend managing patients with stroke-like episodes at the intensive care unit in the following settings:

○ generalised, convulsive status epilepticus;
○ intrusive, frequent focal motor seizures with breakthrough generalised seizures which fail to respond to IV AEDs (and titration of usual maintenance AEDs);
○ severe encephalopathy (with breakthrough focal motor or generalised seizures) with a high risk of aspiration;
○ focal motor status epilepticus with retained consciousness failing to respond to benzodiazepine and two IV AEDs.

We recommend midazolam as the first choice of general anaesthetics (GA) agent for treating refractory status epilepticus associated with stroke-like episodes. We acknowledge that there are anecdotal reports in the literature suggesting the prolonged use of propofol, especially in the paediatric population, may increase the risk of propofol infusion syndrome ^37^. However, propofol is not contraindicated in refractory status epilepticus associated with stroke-like episodes. The decision to use propofol should be decided on a case-by-case basis.

Following the initiation of general anaesthesia, continuous EEG monitoring of patients with stroke-like episodes should be performed to ensure that seizure activities have been suppressed, and no breakthrough seizures (including non-convulsive seizures) occur. If this is unavailable, EEG should be performed as soon as possible after induction of anaesthesia, and at regular intervals (at least daily) for the duration of anaesthesia. Burst suppression is the commonly used EEG target of GA agents and should be maintained for at least 48 hours.

### Use of L-arginine

The use of L-arginine is controversial. There is little evidence to support vascular involvement in stroke-like episodes only small-scale open-labelled trials and observational data are available to evaluate the efficacy of L-arginine (with or without co-supplementation of citrulline). We are, therefore, unable to recommend its use during the stroke-like episodes.

Neuropsychiatric symptoms in stroke-like episodes

It is important to consider non-convulsive seizures as the cause of new-onset neuropsychiatric symptoms in stroke-like episodes. Some patients may manifest with excessive anxiety, aggressiveness, agitation or psychosis (auditory or visual hallucination) if stroke-like lesions involve frontal, temporal or limbic lobe. Based on our collective clinical experience, the following anti-psychotic medications appear to be efficacious and safe on managing the acute neuro-psychiatric symptoms: haloperidol, benzodiazepine and quetiapine. Liaison psychiatric service should be consulted to guide assessment, treatment and to monitor the progress.

Monitoring for the development of arrhythmia with the introduction of an antipsychotic drug may be necessary especially in patients with the m.3243A>G mutation, other rare mtDNA point mutations, and a known history of pre-existing pre-excitation syndrome (e.g. Wolff-Parkinson-White syndrome) or cardiomyopathy.

### Fluid management in mitochondrial disease

Maintenance IV fluid should be administered for patients who are at risk of dehydration, especially in those with encephalopathy or those presenting with vomiting due to intestinal pseudo-obstruction. Careful monitoring of fluid and electrolyte balance may be necessary in those patients with low body mass index, cardiomyopathy or chronic kidney disease as part of the multisystem mitochondrial disease.

### Management of lactic acidaemia

Some patients with mitochondrial disease have elevated plasma lactate (serum lactate level: 2.2–5.0 mmol/L with pH > 7.30) and they often respond well to rehydration. A buffering agent such as sodium bicarbonate can be used with care in severe lactic acidosis (pH <7.1) ^38^. However, the management of severe metabolic acidosis should be shared with the intensivist or nephrologist. We do not recommend the use of dichloroacetate on lowering lactic acid in mitochondrial diseases as it has been shown to cause toxic neuropathy ^39^.

### Nutrition

Nasogastric (NG) or nasojejunal (NJ) tube feeding should be considered for sedated/encephalopathic patients whose oral calorific intake is inadequate (for those without a pre-existing gastrostomy tube or other form of percutaneous feeding tube in situ). Since gastric dysmotility is a recognised problem, we suggest regular low volume continuous administration and avoidance of large boluses that may result in vomiting and aspiration. Early consideration of total parenteral nutrition if prolonged fasting is anticipated for patients with refractory IPO. Early consultation with the nutritional team is recommended during admission for stroke-like episodes.

Blood sugar level should be closely monitored, particularly in patients who harbour primary mtDNA pathogenic variants, during an acute stroke-like episode and managed accordingly.

### Management of gut dysmotility

Patients can develop intestinal pseudo-obstruction (IPO) concomitantly with the stroke-like episodes, often in the background of chronic constipation. Gastroparesis and small bowel IPO can be particularly dangerous in patients unable to adequately protect their own airway – such as those with encephalopathy, seizures, or bulbar dysfunction. Prompt recognition of clinical symptoms and radiological findings is crucial so that drainage of the stomach content can be achieved with the insertion of the wide-bore nasogastric tube (‘drip and suck’). Concomitant constipation and/or faecal impaction occurs and should be treated. Bowel resection surgery is very rarely indicated in the management of intestinal pseudo-obstruction ^40^.

Clinicians should be advised that serum lactate is not a reliable marker for sepsis or tissue ischaemia in patients with mitochondrial disease.

### Other treatment considerations

- We do not recommend the following treatments for stroke-like episodes: antiplatelet therapy, ubiquinone, riboflavin and creatinine.
- Clinicians should follow their local guidelines on instigating deep vein thrombosis (DVT) prophylaxis for patients presenting with stroke-like episodes.
- Swallowing assessment should be considered in the following circumstance: encephalopathy, presence of cerebellar dysfunction or other focal deficits that may increase the risk of aspiration pneumonia.
- Clinicians should be advised that lactic acidosis, electrolyte disturbances, anti-epileptic drugs and fluid replacement may exacerbate or unmask pre-existing cardiac conduction defects or ventricular impairment.

### Genetic testing

If a stroke-like episode is suspected in an individual without a pre-existing diagnosis of mitochondrial disease, urgent genetic testing should be considered as this will have implications for the clinical management. We recommend that at least two tissue samples (e.g. blood and urine samples or blood and buccal samples) should be sent for the genetic studies because mtDNA pathogenic variants such as the m.3243A>G mutation (or other) may not be detectable in blood. If the m.3243A>G or other mtDNA variant is detected, their heteroplasmy level in different tissues should be quantified.

Muscle biopsy should be considered after excluding m.3243A>G and *POLG* variants. Whole mtDNA sequencing of non-invasive tissue (such as urine epithelial cells) should be considered if muscle biopsy not possible in patients present with classical stroke-like episodes.

A detailed family pedigree should be obtained and genetic testing should be offered to at-risk individuals where a pathogenic mutation has been identified.

### Prophylactic anti-epileptic medication for stroke-like episodes

We recognise that there is no proven prophylactic medication currently available for mitochondrial stroke-like episodes. However, we would recommend considering a prophylactic use of an anti-epileptic drug in patients harbouring *POLG* recessive pathogenic variants who are deemed at high risk of developing life-threatening stroke-like episodes.

## Conclusion

We have collaboratively devised this guidance for the investigation, acute management and genetic testing of mitochondrial stroke-like episodes. We acknowledge that the majority of the recommendations are not evidence-based due to the inherent challenges of conducting clinical trials in rare diseases. Our recommendations are derived from the consensus approach, based on clinical experience from the leading mitochondrial clinical services of several European countries and the appraisal of current literature. We believe that standardisation of clinical practice is crucial in mitigating the devastating complications associated with stroke-like episodes, and hopefully, will inform more the robust design of therapeutic trial in this challenging condition. There are emerging novel compounds and clinical trials in mitochondrial disorders, and this document will require a regular update to reflect the development of new evidence.

## Data availability

### Underlying data

Figshare Ng *et al.* Consensus-based Statements for The Management of Mitochondrial Stroke-like Episodes. https://doi.org/10.25405/data.ncl.10740917 ^35^.

This project contains individual participant responses collated for the questionnaires pre- and post-workshop.

### Extended data

Figshare: Ng *et al.* Consensus-based Statements for The Management of Mitochondrial Stroke-like Episodes. https://doi.org/10.25405/data.ncl.10740917 ^35^.

This project contains the following extended data:

Supplemental Table 1. Summary of the agreement (consensus) achieved in the first questionnaire.

Supplemental Table 2. Summary of the agreement (consensus) achieved in the second questionnaire.

Supplemental Table 3. Pre- and post-workshop weighted scores for all statements/recommendations.

Data are available under the terms of the Creative Commons Attribution 4.0 International license (CC-BY 4.0).
