# Supplementary material for: Consensus-based statements for the management of mitochondrial stroke-like episodes
Source: Wellcome Open Res. 2019 Dec 13;4:201. [Version 1] doi: 10.12688/wellcomeopenres.15599.1 (PMC7014928; doi:10.12688/wellcomeopenres.15599.1)
Supplement: Peer review history

# Peer review: Reviewer response for version 1

**DOI:** 10.21956/wellcomeopenres.17083.r37641

**Published:** 2020-02-11

**Authors:** Cornelia Kornblum

**Affiliations:** 1 Department of Neurology, University Hospital of Bonn, Bonn, Germany; 2 Center for Rare Diseases Bonn (ZSEB), University Hospital of Bonn, Bonn, Germany

In this paper, a group of 12 authors from the UK, Finland, Germany, Italy, and Norway published consensus-based statements for the management of mitochondrial stroke-like episodes. Stroke-like episodes are well-known severe neurological manifestations associated with some forms of mitochondrial disorders due to mitochondrial DNA mutations or nuclear gene defects. The underlying mechanisms still remain unclear, however several hypotheses exist regarding i) the role of vascular pathology including impaired cerebral autoregulation, ischemia and/or hyperperfusion ("vascular theory“), or ii) neuronal hyperexcitability including ictal activity and focal or generalised, convulsive or non-convulsive status epilepticus. Limitations in the understanding of the fundamental mechanisms of stroke-like episodes are reflected by controversies surrounding the management of these serious features.

 The authors aimed to discuss the definition, diagnostic procedures and best treatment of stroke-like episodes in order to give consensus-based recommendations for the acute management in the clinical practice.

 Since conducting clinical trials in rare diseases is challenging and especially complicated in mitochondrial disorders, the majority of the given recommendations are not evidence-based but derive from a consensus approach based on clinical experience from leading mitochondrial disease experts while also considering the current literature.

 The authors applied the modified Delphi method consisting of two rounds of email questionnaires with lists of statements and a face-to-face meeting in the UK. The first questionnaire contained 88 questions and was subsequently modified and devised during the face-to-face meeting resulting in a questionnaire finally containing 77 questions. All underlying data including anonymised individual participants‘ responses are available in the supplementary material of the paper. Representatives from patient and charity organisations attended the face-to-face workshop and participated in the discussion.

 Consensus on a new definition of mitochondrial stroke-like episodes was achieved stating that the episodes are driven by neuronal hyperexcitability and epileptic seizures. This is in contrast to the hypothesis of a primarily vascular, ischemic origin that has not been convincingly proven to date. Further, the authors developed refined diagnostic criteria mainly based on clinical features, brain MR-imaging and/or EEG findings. Most importantly, recommendations for the acute management of stroke-like episodes and the treatment of immediate sequelae are given in a detailed and valid way. The recommendations stress the importance of early and aggressive seizure management. Though final consensus was achieved regarding definition and management of stroke-like episodes, the authors conclude that a prospective, multi-centre, randomised controlled trial is still required for evaluating the efficacy of any compound on modifying the course of these devastating disease features.

 The paper addresses an important and clinically relevant aspect of acute complications of specific mitochondrial disorders. The topic is highly relevant for mitochondrial disease experts, general neurologists, neurovascular specialists, intensivists but also for general practitioners. These consensus-based recommendations deal with a variety of difficult questions and help to handle common challenges in the management of stroke-like epsiodes that often represent fatal complications. It is of particular importance to give substantiated and best medical advice to clinicians in charge of these patients. Mitochondrial diseases are not yet curable, and stroke-like episodes cannot be fully prevented or satisfactorily treated to date. Thus, a most standardised approach to these devastating recurrent episodes is mandatory.

 The applied modified Delphi method is a frequently used technique to study the group opinion of experts in order to develop recommendations or guidelines in medical fields where there is little evidence, insufficient or conflicting data. The authors group is dominated by UK experts, however includes mitochondrial specialists from four European countries. Getting into detail of the addressed statements and clinical aspects, the recommendations precisely reflect the common clinical practice and procedures well-established in European mitochondrial expert centers.

I confirm that I have read this submission and believe that I have an appropriate level of expertise to confirm that it is of an acceptable scientific standard.

# Peer review: Reviewer response for version 1

**DOI:** 10.21956/wellcomeopenres.17083.r37390

**Published:** 2020-01-17

**Authors:** Costanza Lamperti

**Affiliations:** 1 Unit of Medical Genetics and Neurogenetics, Fondazione IRCCS Istituto Neurologico Carlo Besta, Milan, Italy

This is an interesting paper in which a group of 12 clinician experts in mitochondrial disease, coming mostly from UK and from 4 European countries, discussed the treatment of acute stroke like episodes on MELAS patients using a modified Delphi method, in order to produce consensus guidance for the acute management of stroke-like episodes and commonly associated complications.

 Consensus on a new definition of mitochondrial stroke-like episodes as epileptic encephalopathy was achieved revising both diagnostic criteria and guidelines for the management. The group developed diagnostic criteria based on clinical features, neuroimaging and/or electroencephalogram findings. Guidelines for the management of stroke-like episodes were agreed focused on epileptic status. A prospective, multi-centre, randomized controlled trial is required for evaluating the efficacy of any compound to modify the outcome of stroke-like episodes.

 The modified Delphi method is the most used method to validate situations where there is little evidence, where data are incomplete or not readily applicable to the area of interest, or where data are extensive but contradictory. Methodology is correct and the best available one. My unique concern is that the method needs 70% agreement but, actually, 72% of participants are form UK and most of them from the same center. This could lead to a predominance of UK clinical practice and experience

 Nevertheless the clinical approach that has been agreed in the consensus reflect the common sense and the good clinical practice .

 The paper is useful, faces an important aspect of mitochondrial disease and gives a useful guideline for stroke-like episode in MELAS. I agree with the authors that standardization of clinical practice is crucial in mitigating the devastating complications associated with stroke-like episodes, and that could be a starting point for further multicenter randomized studies in order to provide more robust design of therapeutic trials on this condition, that is still not possible to prevent and manage to an adequate level, and often lead to patient deaths.

I confirm that I have read this submission and believe that I have an appropriate level of expertise to confirm that it is of an acceptable scientific standard.
